# It's About Time—Breathing Dynamics Modulate Emotion and Cognition

**DOI:** 10.1111/psyp.70149

**Published:** 2025-09-11

**Authors:** Josh Goheen, Yasir Çatal, Imola MacPhee, Tyler Call, Cameron Carson, Reem Ali, Rabeaa Khan, Kareen Weche, John A. E. Anderson, Georg Northoff

**Affiliations:** ^1^ Department of Cognitive Science Carleton University Ottawa Ontario Canada; ^2^ The Royal and the University of Ottawa's Institute of Mental Health Research Ottawa Ontario Canada; ^3^ Department of Psychology Carleton University Ottawa Ontario Canada

**Keywords:** anxiety, breathing, cognition, dynamics, neurovisceral integration model

## Abstract

The breathing rate, phase, and amplitude have been shown to track changes in emotional states such as anxiety and cognitive performance in tasks that involve perception, attention, and short‐term memory. It is common practice to characterize breathing by using a block average breathing rate, phase, or amplitude. While these features are useful for measuring the central tendencies of breathing, they do not capture the structure of the patterns of change in its activity over time (i.e., breathing dynamics) whose relationship with affective and cognitive processes remains unclear. To fill this knowledge gap, we characterized breathing dynamics by a set of measures that capture the breathing signal's rate and amplitude central tendency, variability, complexity, entropy, and timescales. Then, we conducted a principal components analysis and demonstrated that these metrics capture similar, yet distinct features of the breathing rate and amplitude time series. Next, we showed that breathing dynamics change across rest and task conditions, suggesting they may be sensitive to changes in behavioral states. Finally, using multivariate analyses, we demonstrated that breathing complexity and entropy in the resting state are strongly and positively correlated with anxiety levels, while breathing variability in the task state is strongly and negatively associated with working memory performance. Our findings extend the current understanding of how breathing is associated with affective and cognitive processes by highlighting the key role of dynamics in that relationship.

## Abstract 2

Breathing modulates both brain activity and behavioral performance, yet its dynamic features remain understudied. We show the relative contribution of different breathing metrics to the overall variance of the breathing signal in the resting state. Next, we show that breathing dynamics contribute in different ways to subjects' anxiety levels and working memory performance. Our work extends the Neurovisceral Integration model by indicating that dynamics may be key in mediating the impact of breathing on emotion and cognition through the brain.

## 1 Introduction

Scientists and mental health practitioners have a long tradition of using breathing regulation to modulate psychological states (Fincham et al. 2023; Zaccaro et al. 2018). For instance, the role of breathing regulation has been studied in various meditation and stress regulation techniques (Ashhad et al. 2022; Balban et al. 2023; Del Negro et al. 2018; Kox et al. 2014; Russo et al. 2017; Tatschl et al. 2020). Despite the known relationship between breathing and psychological states, the link between spontaneous breathing and cognitive and affective processes remains poorly understood. Addressing this knowledge gap was the main goal of our study.

The breathing rhythm is associated with affective and cognitive functions. This is evident in anxiety disorders (AD) where patients with AD present with shallower, more rapid breathing and an increased rate of sighing while at rest compared to their healthy counterparts (Giardino et al. 2007; Meuret and Ritz 2010; Ritsert et al. 2022; Severs et al. 2022; Vlemincx et al. 2017). Beyond its relationship with affective processes like anxiety, breathing also appears to play a role in cognitive functions. For instance, engaging in a cognitive task that involves attentional control or short‐term memory increases the breathing rate and decreases the breathing amplitude with medium to large effect sizes compared to the resting state; this reflects subtle changes in the breathing rhythm under different cognitive loads (Goheen et al. 2024; Grassmann et al. 2016; Jaiswal et al. 2019).

Are there features of breathing that are meaningful for cognitive performance? Emerging evidence suggests that performance on short‐term memory, attentional control, and perceptual sensitivity tasks varies systematically when stimuli are presented across different phases of the breathing cycle (Nakamura et al. 2023; Saltafossi et al. 2025; Zaccaro et al. 2025). For example, Johannknecht and Kayser (2022) explored the association of the breathing phase relative to stimulus presentation and responses on short‐term memory, emotional discrimination, sound detection, and visual motion discrimination performance. The authors found that the inhalation phase of the breathing cycle was generally associated with faster responses compared to exhalation, with the median values for the effects falling between 20 and 50 ms. Similarly, a 2025 study by Braendholt and colleagues found that responses on a visual discrimination task were faster when button clicks coincided with inspiration versus expiration, with similar effect sizes (Brændholt et al. 2025). Moreover, during short‐term memory paradigms when retrieval spans the exhalation–inhalation transition, reaction times are approximately 20% slower and accuracy rates decline by around 10% as compared to rates not spanning the exhalation–inhalation transition (Nakamura et al. 2018, 2022, 2023).

Together, the current literature suggests that different dimensions of the breathing rhythm are strongly coupled to affective and cognitive processes. Recent work has provided a computational framework for understanding how breathing, neuronal activity, and behavioral functions like emotional processing, attentional control, and memory may fluctuate as a function of each other (Allen et al. 2023; Brændholt et al. 2023; Criscuolo et al. 2022; Harting et al. 2025; Kluger, Allen, and Gross 2024; Kluger, Gross, and Keitel 2024; Kozma et al. 2022; Tort et al. 2018, 2025; Varga and Heck 2017). This framework suggests a key role of patterns of activity within the breathing signal itself (i.e., breathing dynamics) in mediating its relationship with affective and cognitive processes; this remains to be explored directly, however (Oku 2022; Soni and Muniyandi 2019). By considering different dimensions of how the breathing signal fluctuates over time, we move towards a framework whereby spontaneous breathing dynamics may provide a physiological window into affective and cognitive functions.

**FIGURE 1:**
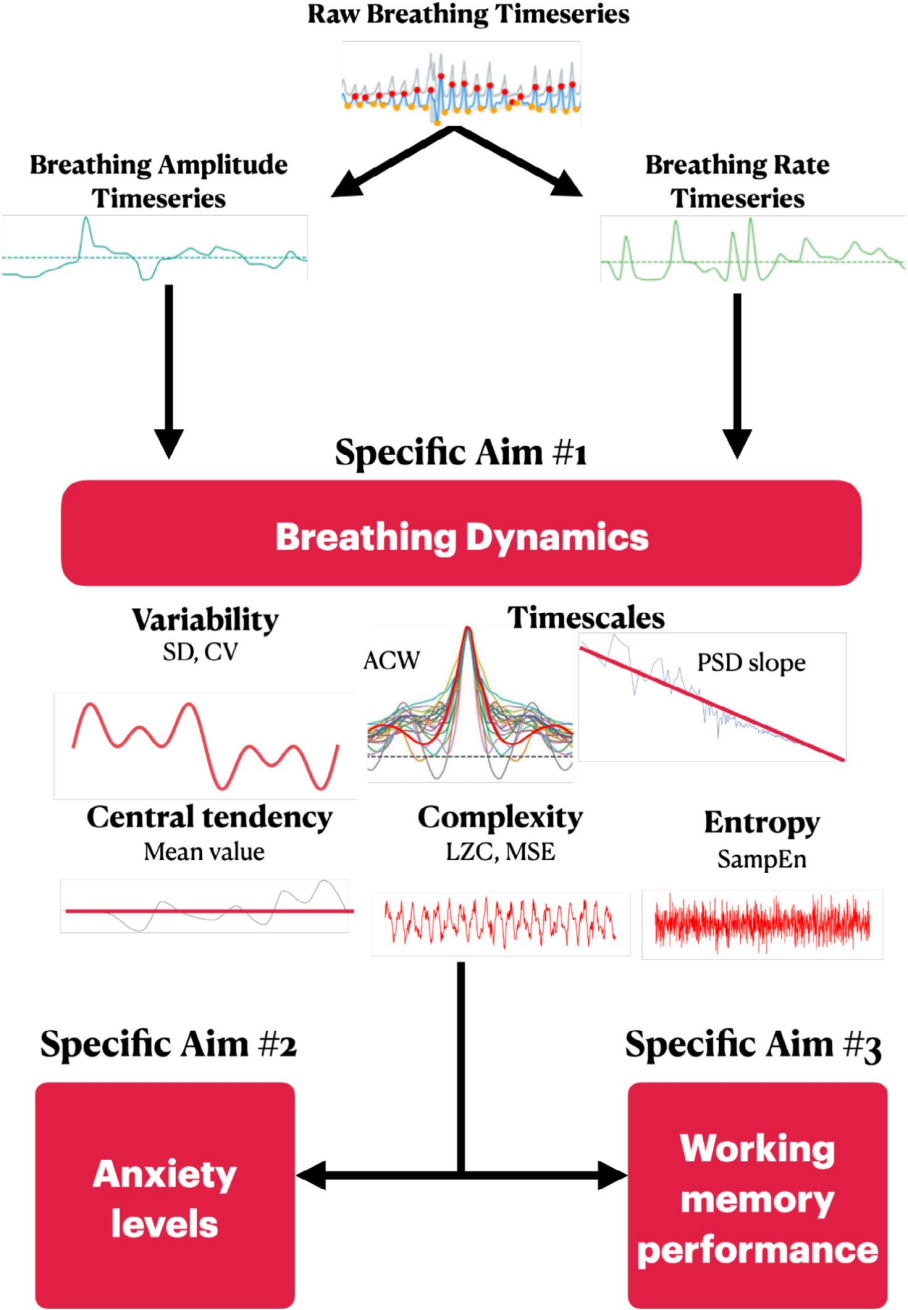
Overview of the paper. Our first aim was to characterize breathing using a set of features which specify breathing's intrinsic patterns of activity over time (i.e., breathing dynamics). Our second specific aim consisted of relating breathing dynamics to emotion and specifically anxiety levels as measured by the Beck Anxiety Inventory (BAI). Our third aim is to relate breathing dynamics to cognitive performance as measured by efficiency during a working memory task (accuracy/reaction time). ACW = autocorrelation window, CV = coefficient of variation, LZC = Lempel‐Ziv complexity, MSE = multiscale entropy, PSD slope = power spectral density slope, SampEn = sample entropy, SD = standard deviation.

Breathing dynamics can be captured using a range of time series measures. For instance, breathing variability can be measured by calculating the standard deviation (SD) and coefficient of variation (CV) of the breathing signal's time series. SD indicates overall variability, and CV provides a standardized measure of variability (Khorana et al. 2023; Ospina and Marmolejo‐Ramos 2019). Beyond a point estimate of variability, physiological signals like breathing or brain activity carry an inherent temporal structure (Golesorkhi et al. 2021; Huang et al. 2016). The autocorrelation window (ACW) quantifies the degree of similarity between a signal and a lagged version of itself over shorter and longer time intervals (Honey et al. 2012; Wolman et al. 2023). Closely related but in the frequency domain, the power spectrum slope (PSD slope) shows how power is distributed across a signal's frequency spectrum (Northoff 2024). Furthermore, physiological signals such as breathing are inherently complex (B. J. West et al. 2023). Lempel‐Ziv complexity (LZC) evaluates the degree to which the signal can be compressed, that is, the size of the pattern space that is necessary to explain a signal, which indicates how complex that signal is (Amigó et al. 2004; Lempel and Ziv 1976; Zhang et al. 2009). Sample entropy (SampEn) quantifies the irregularity of a signal by measuring the degree to which present time points can predict future ones (Richman and Moorman 2000). Multiscale entropy (MSE) applies a coarse‐graining procedure to the time series, analyzing how patterns in sample entropy persist in the data across shorter and longer timescales; this allows the MSE to measure the complexity of the signal (Costa et al. 2002, 2005; Hutcheon et al. 2023; Lee and Choi 2018). By considering these measures, we can capture the temporal structure of the patterns of change in the breathing signal over time (i.e., breathing dynamics), potentially providing a nuanced understanding of how breathing is associated with affective and cognitive processes (Figure 1).

Our first specific aim was to characterize the breathing signal's dynamics. To do this, we considered the breathing rate and amplitude time series and computed the metrics described above. We hypothesized that these metrics should identify related yet distinct aspects of the breathing signals' dynamics and change based on testing conditions. To test this assumption, we determined the relative contribution of each metric to the overall variance in the breathing signal in the resting state using a principal components analysis (PCA). Then, we compared breathing dynamics during rest and task conditions using paired samples *t*‐tests. Together, these would indicate if breathing dynamics are sensitive to changes in behavioral states.

Our second specific aim was to relate breathing dynamics to emotion and specifically anxiety as measured by the Beck Anxiety Inventory (BAI). Our third aim was to relate breathing dynamics to cognitive performance as measured by behavioral efficiency during a working memory task. Prior work shows that breathing rate increases while the breathing amplitude decreases in anxious states (Dias et al. 2024; Giardino et al. 2007; Masaoka et al. 2001; Masaoka and Homma 1997, 2001). While the breathing rate increases and breathing amplitude decreases during cognitive tasks as well, it is noteworthy that the variability and periodicity of breathing track cognitive load and task engagement, suggesting that the set of features linking breathing to affective and cognitive processes, respectively, may overlap but be distinct (Goheen et al. 2024; Grassmann et al. 2016; Kluger, Gross, and Keitel 2024; Melnychuk et al. 2021). Therefore, by applying a multivariate analysis using partial least squares correlation (PLSC), we hypothesized that we would find subprofiles of breathing dynamics which relate to cognitive (i.e., working memory performance) and affective processes (i.e., anxiety levels) in distinct ways.

Together, our findings show that the central tendency, variability, entropy, complexity, and timescales of the breathing signal take on different roles in mediating the effect of breathing on emotion/anxiety and cognition/working memory. Our findings extend the current understanding of how breathing is related to affective and cognitive processes by highlighting the key role of dynamics in that relationship.

## 2 Results

### 2.1 Dynamics Quantify Distinct Features of the Breathing Signal's Intrinsic Patterns of Activity Over Time

**FIGURE 2:**
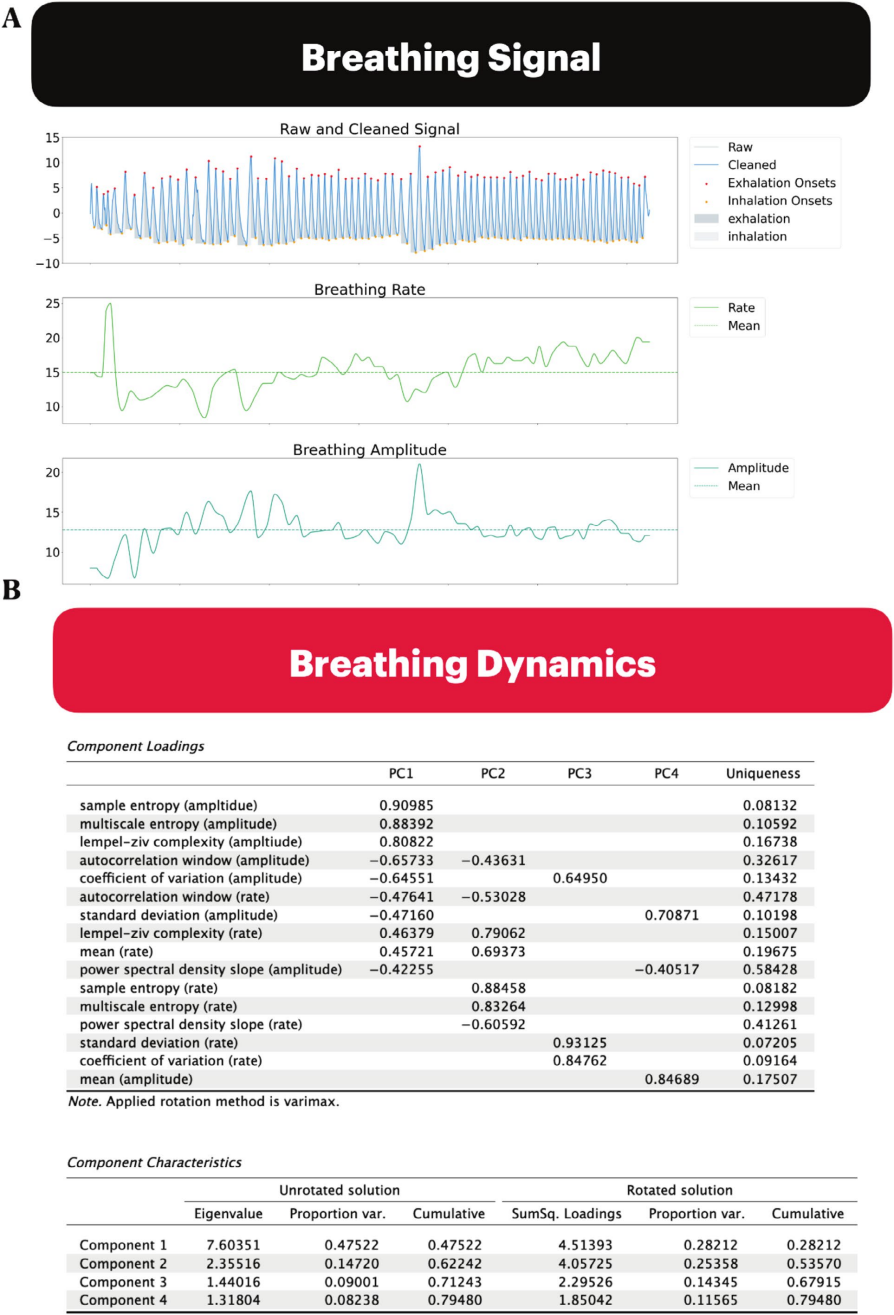
(A) Single subject resting state breathing recording. (B) PCA of breathing dynamics during the resting state (*n* = 210). Loadings < 0.4 are not shown in panel B.

Figure 2A presents a sample breathing recording from a single subject in the resting state. The top, middle, and bottom panels represent the raw breathing vector, rate, and amplitude time series, respectively. Visual inspection of the breathing signal indicates that its rate and amplitude are constantly fluctuating over time. Our work investigates if there are specific patterns of change and, thus, information, embedded within the breathing signal that relate to emotion and cognition.

We first investigated the breathing signal in the resting state for two reasons: (1) to verify that our measures were capturing distinct aspects of the breathing signal's dynamics, and (2) to determine the relative contribution of each metric to the overall variance in the breathing signal. To do this, we conducted a principal components analysis (PCA) of the dynamic measures extracted from the breathing signal's rate and amplitude time series. Our analysis considered the breathing rate and amplitude time series central tendency (mean values), variability (SD, CV), complexity (LZC, MSE), entropy (SampEn), and timescales (ACW, PSD slope). Figure 2B presents the component loadings. The first four PCs explained 28.2%, 25.3%, 14.3%, and 11.6% of the total variance, respectively. PC1 was primarily characterized by strong positive loadings from SampEn (amplitude; 0.91), MSE (amplitude; 0.88), and LZC (amplitude; 0.81). PC2 was dominated by strong positive loadings from SampEn (rate; 0.88), MSE (rate; 0.83), and LZC (rate; 0.79). PC3 captured mainly the SD (rate: 0.93) and CV (rate: 0.85). Finally, PC4 was broadly defined by mean (amplitude, 0.85) and SD (amplitude, 0.71).

Collectively, these findings indicate that PC1 (28.2% of the total variance) primarily detected breathing dynamics in the amplitude time series, while PC2 (25.3% of the total variance) is picking up on the rate time series. Moreover, entropy and complexity measures seem to explain the most variance in the breathing signal indicated by SampEn, MSE, and LZC, having the strongest loadings onto PC1 and PC2.

### 2.2 Breathing Dynamics Are Modulated Across Rest and Task Conditions

Do breathing dynamics change under different cognitive demands? To address this question, we conducted paired samples *t*‐tests comparing breathing patterns across rest and task states (see Table 1). Engaging in a working memory task (visual *N*‐back—see methods for full details of the task) was associated with significant alterations in breathing's central tendencies, including an increase in the mean breathing rate *t*(50) = −6.49, *p* < 0.001, *d* = −0.91, and a decrease in the mean amplitude *t*(50) = 6.92, *p* < 0.001, *d* = 0.97, indicating large effect sizes.

**TABLE 1:** Engaging in a working memory task modulates breathing dynamics (*n* = 51).

Paired samples *T*‐Tes				
Rest	Task	*t*	*p* (adjusted)	Cohen's *d*
Mean (rate)	Mean (rate)	−6.48587	1.53 × 10^−7^***	−0.90820
Mean (amplitude)	Mean (amplitude)	6.92503	4.19 × 10^−8^ ***	0.96970
Standard deviation (rate)	Standard deviation (rate)	−5.50576	3.39 × 10^−6^***	−0.77096
Standard deviation (amplitude)	Standard deviation (amplitude)	1.85969	0.085	0.26041
Coefficient of variation (rate)	Coefficient of variation (rate)	−1.82117	0.085	−0.25501
Coefficient of variation (amplitude)	Coefficient of variation (amplitude)	−2.46755	0.023*	−0.34553
Autocorrelation window (rate)	Autocorrelation window (rate)	3.39784	0.0021**	0.47579
Autocorrelation window (amplitude)	Autocorrelation window (amplitude)	3.06423	0.0051**	0.42908
Power spectral density slope (rate)	Power spectral density slope (rate)	5.88710	1.05 × 10^−6^***	0.82436
Power spectral density slope (amplitude)	Power spectral density slope (amplitude)	0.25811	0.797	0.03614
Lempel‐Ziv complexity (rate)	Lempel‐Ziv complexity (rate)	−4.76452	4.57 × 10^−5^ ***	−0.66717
Lempel‐Ziv complexity (amplitude)	Lempel‐Ziv complexity (amplitude)	−3.92801	4.62 × 10^−4^***	−0.55003
Multiscale entropy (rate)	Multiscale entropy (rate)	−11.39142	2.68 × 10^−14^***	−1.59512
Multiscale entropy (amplitude)	Multiscale entropy (amplitude)	−7.81498	2.57 × 10^−9^ ***	−1.09432
Sample entropy (rate)	Sample entropy (rate)	−4.44240	1.00 × 10^−4^***	−0.62206
Sample entropy (amplitude)	Sample entropy (amplitude)	−1.24799	0.232	−0.17475

*Note:* The *p*‐values presented are corrected for multiple comparisons using the Benjamini‐Hochberg false discovery rate method. Raw values that were used for statistical comparisons presented in this table can be found in Table S1. Student's *t*‐test. *, **, *** indicates the statistical significance of *p*‐values when *p < 0.05, **p < 0.01, ***p < 0.001 respectively.

Breathing variability also significantly increased from rest to task when measured with the standard deviation (SD) of the breathing rate *t*(50) = −5.50, *p* < 0.001, *d* = −0.77, and coefficient of variation (CV) of the breathing amplitude time series *t*(50) = −2.47, *p* < 0.05, *d* = −0.35, indicating medium and small effect sizes, respectively.

Furthermore, compared to rest, breathing signals during task led to a restructuring of the breathing signals timescales, evidenced by a decrease in the autocorrelation window (ACW) with small effect sizes for both the rate *t*(50) = 3.40, *p* < 0.01, *d* = 0.48, and amplitude time series *t*(50) = 3.06, *p* < 0.01, *d* = 0.43. These findings were corroborated by a decrease in the power spectral density slope (PSD slope) in the breathing rate time series with a large effect size *t*(50) = 5.88, *p* < 0.001, *d* = 0.82. Together, the decrease in ACW and PSD slope indicates an overall shift of the breathing signal towards faster frequencies.

Breathing complexity increased from rest to task as evidenced by increases in Lempel‐Ziv complexity (LZC) with medium effect sizes for both the rate *t*(50) = −4.76, *p* < 0.001, *d* = −0.67, and amplitude time series *t*(50) = −3.92, *p* < 0.001, *d* = −0.55. These findings were supported by increases in multiscale entropy (MSE) from rest to task with large effect sizes for both the amplitude *t*(50) = −7.81, *p* < 0.001, *d* = −1.60, and rate time series *t*(50) = −11.39, *p* < 0.001, *d* = −1.1. Similarly, sample entropy (SampEn) for the breathing rate exhibited marked increases from rest to task *t*(50) = −4.44, *p* < 0.001, *d* = −0.62, indicating a medium effect size.

### 2.3 Breathing Dynamics Relate to Emotion and Cognition in Different Ways

**FIGURE 3:**
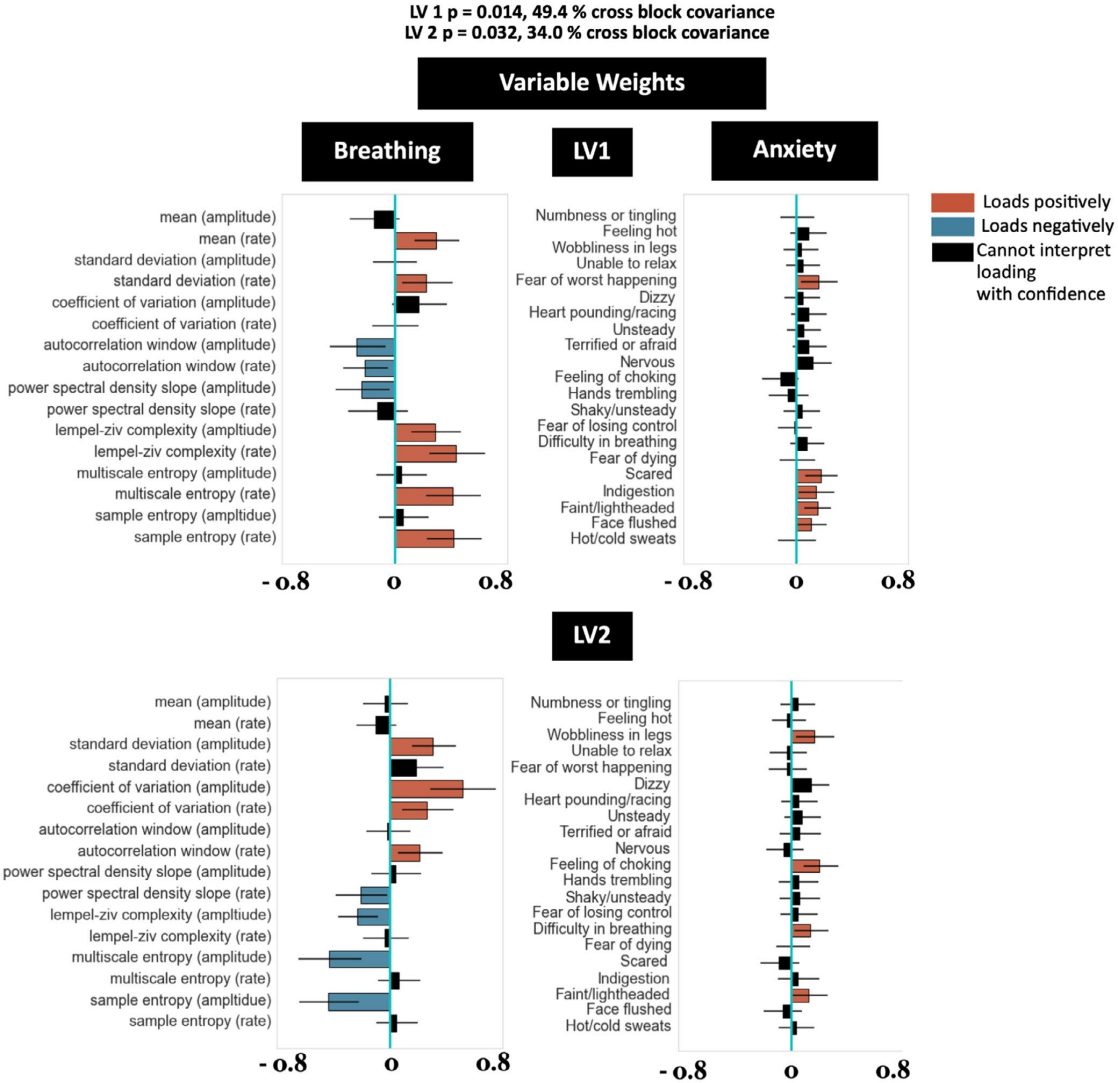
Variable weights from the partial least squares correlation (PLSC) demonstrating the relationship between breathing dynamics in the resting state and emotion as measured by anxiety levels (i.e., BAI scores) (*N* = 210). Whiskers on the bars represent the 95% confidence intervals of the variable weights assessed from 1000 bootstrap samples.

We next asked whether breathing dynamics are related to emotions like anxiety. To test for this, we compared breathing dynamics in the resting state and anxiety levels using the Beck Anxiety Inventory (BAI). Our findings from the partial least squares correlation (PLSC) analysis between breathing and anxiety levels identified two key latent variables (LVs) (*p*'s < 0.05) that explained 49.4% and 34.0% of the cross‐block covariance, respectively. When looking at the features which loaded most strongly into the latent variable space, the first LV shows breathing signal complexity (MSE, LZC) and entropy (SampEn) scores correlated strongly and positively with levels of anxiety. The second LV shows low signal complexity (MSE, LZC) and entropy (SampEn) scores when combined with high signal variability (SD, CV), which also correlated strongly and positively with anxiety levels (Figure 3). Our investigations suggest that there are multiple sub‐profiles of breathing that relate to anxiety levels. Importantly, we can see differential contributions of the distinct metrics of the breathing signal's dynamics to the explanatory variance of anxiety levels.

**FIGURE 4:**
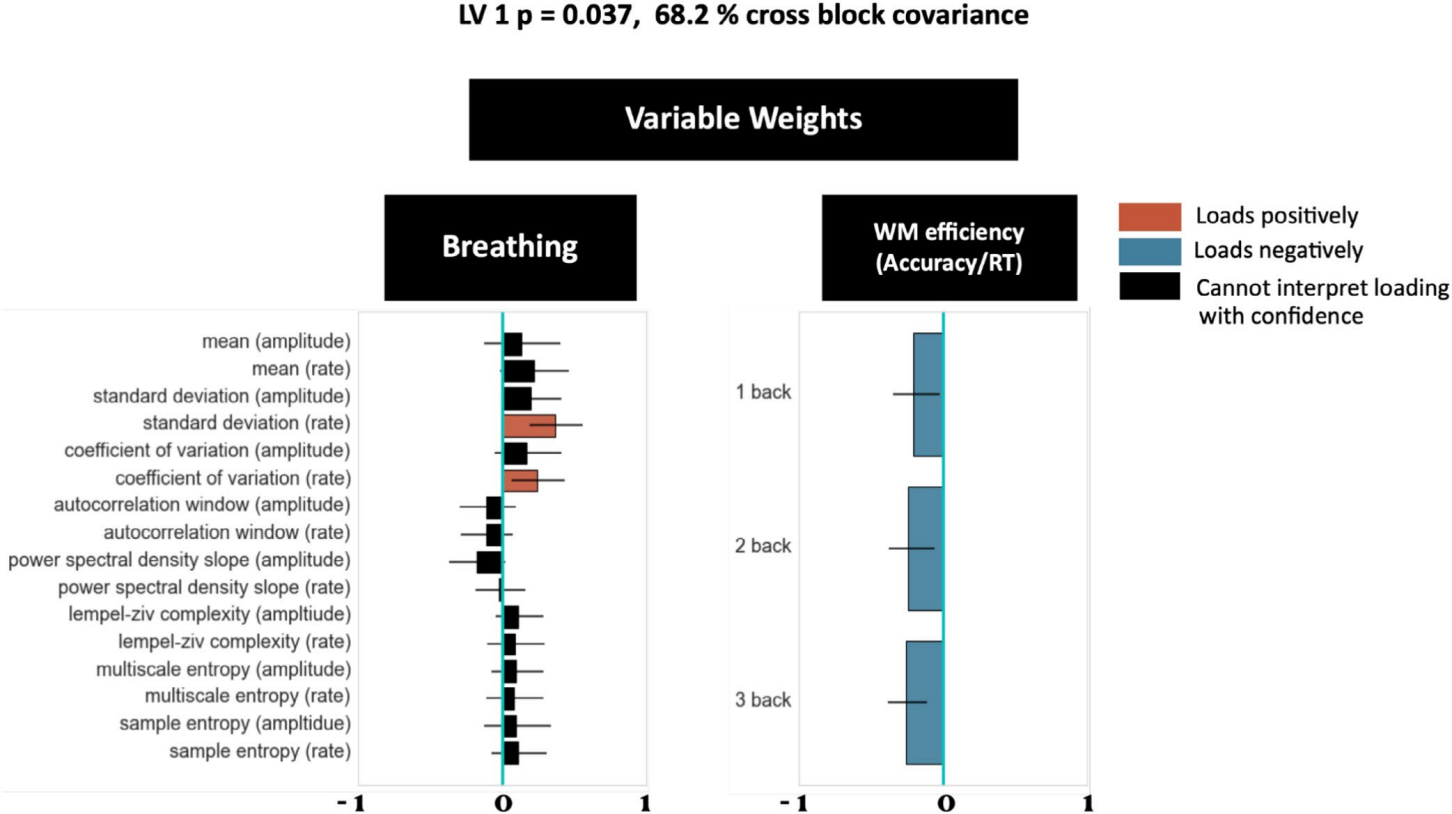
Variable weights from the partial least squares correlation (PLSC) demonstrating the relationship between breathing dynamics in the task state and cognition as measured by working memory efficiency (accuracy/reaction time) across increasing load conditions (i.e., 1‐back, 2‐back, and 3‐back; *N* = 51). Whiskers on the bars represent the 95% confidence intervals of the variable weights assessed from 1000 bootstrap samples [Random hold out cross validation yielded less reliable results compared to standard PLSC resampling methods, particularly with breathing and anxiety (*r* = 0.024) compared to working memory (*r* = 0.183). Future research will need to expand on our descriptive model (i.e., PLSC) and move into a predictive space by collecting a larger sample and applying regression‐based models (i.e., PLS regression)].

Next, we questioned whether breathing dynamics are also related to cognitive functions like working memory. To test for this, we compared breathing dynamics during the task state with performance on an *N*‐back task. Our findings in the PLSC analysis between breathing and working memory performance identified one significant LV (*p* < 0.05), which explained 68.2% of the total cross‐block covariance. When looking at the features that loaded most strongly into the latent variable space, the LV showed higher coefficient of variation (CV) and standard deviation (SD) scores correlated strongly and negatively with working memory performance, regardless of task difficulty (Figure 4). Our investigations suggest that signal variability plays a key role in linking breathing to working memory performance. Notably, we see that different metrics of the breathing signal's dynamics relate to working memory and anxiety levels.

To verify if anxiety levels moderated the relationship between breathing dynamics and working memory performance, we performed an interaction analysis using the breathing variables which loaded reliably and strongly into the latent variable space in the analysis between breathing dynamics and working memory performance (i.e., SD and CV) using the interactions package in R (Long 2024). We found that anxiety levels moderate the relationship between breathing dynamics and working memory performance; however, this relationship is relatively weak and only holds for the coefficient of variation of the breathing rate at lower levels of anxiety (Figure S6).

Finally, we replicated our analyses between breathing and anxiety levels as well as breathing and working memory performance using only the mean breathing rate and amplitude values. No reliable LVs were found for either analysis, highlighting the importance of using dynamics in relating breathing to affective and cognitive processes (Figures S7 and S8).

## 3 Discussion

### 3.1 Dynamics Are Relevant for Explaining the Breathing Signal

The first specific aim of our work was to characterize the breathing signal using a set of metrics which capture distinct aspects of its dynamics. Our investigation considered the breathing rate and amplitude time series' central tendency (mean values), variability (SD, CV), complexity (LZC, MSE), entropy (SampEn), and timescales (ACW, PSD slope) (Northoff 2024).

We first performed a PCA of breathing dynamics in the resting state. This verified that we were capturing distinct aspects of the breathing signal's dynamics and determining the relative contribution of each metric to the overall variance in the breathing signal between subjects. Overall, the PCA revealed a broad range of variable loadings and latent structures in the breathing signal, suggesting that these metrics capture similar yet distinct features of breathing's intrinsic patterns of activity over time (Figure 2).

Furthermore, our investigations revealed clear increases in the breathing signal's mean rate, variability, entropy, and complexity and a decrease in its mean amplitude and timescales from rest to task (Table 1). These adaptations in breathing dynamics indicate that breathing dynamics are shaped by the task, a finding that accords with previous work (Goheen et al. 2024; Lakatos et al. 2019; Potts et al. 2005). In combination with the results from the PCA, the modulation of breathing dynamics by the task suggests that breathing dynamics are sensitive to changes in behavioral states.

Grassmann et al. (2016) conducted a systematic review to evaluate how breathing patterns change in response to changes in cognitive load. The authors reported increases in the breathing rate and a decrease in the breathing rate's autocorrelation under various cognitive demands (i.e., attentional control, mental arithmetic, short‐term memory, etc.), mirroring our observations (Grassmann et al. 2016). However, unlike Grassmann et al., who found overall breathing variability (i.e., CV) largely unchanged, our results indicate significant increases in both variability and complexity measures (Table 1). This difference may reflect that Grassmann et al. aggregated over a diverse set of cognitive tasks, whereas our focus was specifically on a working memory paradigm. In addition, out of the six studies in their review that utilized memory‐specific paradigms, none of them reported how task engagement affected breathing variability, which potentially further explains the discrepancy between our findings and those of Grassmann et al. (2016).

### 3.2 Breathing Dynamics Are Relevant for Affective and Cognitive Processing in the Brain

Although we do not assess neuronal activity directly in this study, our findings raise important questions about the neural substrates which could link spontaneous breathing to emotional and cognitive processes. The neurovisceral integration model (NVIM) posits that cognitive performance and emotional regulation depend on the ability of the central autonomic network (CAN) to regulate vagal tone, with greater vagal control supporting better adaptability to changing cognitive and emotional demands (Scott et al. 2021; Thayer et al. 2009; Thayer and Lane 2000). One key physiological process interacting with vagal tone and activity in the CAN is breathing (Laborde et al. 2022). More specifically, breathing inhalation increases activity within the sympathetic arm of the autonomic nervous system (ANS), and exhalation increases activity within the parasympathetic arm. This highlights a see‐saw‐like relationship between breathing, vagal tone, and, indirectly, the CAN (Cho et al. 2023; Stone et al. 2020). These observations point to a temporally structured relationship between breathing and central autonomic functioning, which could explain our findings.

Breathing is tightly coupled with neural oscillations in CAN regions across multiple frequency bands (Dias et al. 2024; Folschweiller and Sauer 2021, 2023; Herrero et al. 2018; Jelinčić et al. 2022; Karalis and Sirota 2022; Kluger et al. 2025; Kluger and Gross 2021; Kozma et al. 2022; Maric et al. 2020; Nguyen Chi et al. 2016; Noble and Hochman 2019; Tort et al. 2025; Yackle and Do 2025). In line with our findings, such breathing‐neuronal coupling, in turn, is posited to shape attentional control, emotional discrimination, and short‐term memory performance, with these modulatory effects typically yielding medium effect sizes (Andrews 2024; Andrews et al. 2025; Ashhad et al. 2022; Brændholt et al. 2024; Heck et al. 2016; Johannknecht and Kayser 2022; Kluger et al. 2021; Melnychuk et al. 2018; Nakamura et al. 2018, 2022, 2023; Perl et al. 2019; Saltafossi et al. 2025; Zelano et al. 2016). Therefore, in the context of the NVIM, breathing dynamics could reflect meaningful peripheral associations with brain function, potentially mediating the relationship between spontaneous breathing and behavior (Goheen et al. 2023, 2024; Harting et al. 2025; Kluger et al. 2021; Kluger, Gross, and Keitel 2024; Kluger and Gross 2021; Tort et al. 2025). Despite the growing body of evidence linking breathing to vagal tone, brain activity in the CAN, and cognitive/affective functions, the exact features that bridge these levels over time within the context of the NVIM are still unclear.

Traditionally, the Neurovisceral Integration Model has emphasized heart rate variability as a proxy for the regulatory capacity of the central autonomic network over the autonomic nervous system in a univariate framework (de Oliveira Matos et al. 2020; Thayer et al. 2009; Thayer and Lane 2000; Tupitsa et al. 2022). This view, while valuable, has ignored the contribution of the breathing signal. Our work not only extends the NVIM to breathing but does so using a multivariate approach. Multivariate statistics distill a vast array of potentially relevant breathing metrics into a coherent set of components which may converge with an outcome measure of interest. Thus, by considering breathing dynamics in a multivariate framework, our approach yields a novel view of the association between breathing and behavior in the context of the NVIM.

We found strong correlations between breathing dynamics and behavior. For example, the first LV in our PLS analysis about working memory explained 68.2% of the total cross‐block covariance and revealed that high breathing coefficient of variation (CV) and standard deviation (SD) scores during the task were the strongest predictors of lower working memory performance regardless of task difficulty (Figure 4). Our results are in line with the idea that signal variability plays a key role in linking breathing and working memory performance.

There seems to be an even closer connection between breathing dynamics and anxiety. For instance, the first LV in our PLS analysis regarding anxiety explained 49.4% of the total cross‐block covariance and revealed that high breathing Lempel‐Ziv complexity (LZC), multiscale entropy (MSE), and sample entropy (SampEn) scores in the resting state were the strongest predictors of higher anxiety levels. The second LV in our PLS analysis regarding anxiety explained 34% of the total cross‐block covariance and revealed that low signal complexity (MSE, LZC) and entropy (SampEn) scores, when combined with high signal variability (SD, CV) scores, also strongly predicted higher anxiety levels (Figure 3). Our investigations suggest that there are sub‐profiles in the breathing signal's dynamics that relate to different sets of anxiety symptoms. Overall, MSE, LZC, and SampEn loaded strongly onto both LVs, suggesting that signal complexity and entropy may play key roles in linking breathing dynamics to anxiety levels.

Anxiety is characterized by increased complexity and entropy on the psychological level in the subject's experience (Lu et al. 2024). Dysregulation of the breathing signal, as indexed by higher complexity and irregularity, may thus provide a connection between breathing dynamics and affective processes like anxiety. Presupposing the NVIM, one would consequently expect dysregulation to also occur on the neural level in the regions of the CAN whose neural dynamics also may show increased complexity and entropy; that remains to be investigated, however.

Together, our findings are in line with the idea that dynamics may be shared by the peripheral level of the autonomic nervous system, including breathing, the neural level of the brain's CAN, and the psychological level of affective and cognitive processes. If this is the case, one would expect a tight coupling between the features of spontaneous breathing dynamics discussed in this paper and neuronal dynamics in key cognitive and emotional regions. This proposed coupling would align well with the current literature (Allen et al. 2023; Brændholt et al. 2023; Criscuolo et al. 2022; Kluger, Allen, and Gross 2024; Kluger, Gross, and Keitel 2024; Kozma et al. 2022; Saltafossi et al. 2025; Yackle and Do 2025); such shared dynamics as their “common currency” remain to be tested directly, however (Northoff et al. 2020, 2024).

### 3.3 The Road Ahead—An Individualized Approach to Managing Anxiety Using Breathing

Deliberate breathing practices may be an effective tool for regulating cognitive and affective disorders (Goheen et al. 2023; Zaccaro et al. 2018). For example, a recent study meta‐analyzed the available randomized controlled trials on the effect of breathwork on stress and mental health outcomes such as anxiety. Their analysis between stress and breathwork consisted of 12 trials, and their random effects analysis yielded medium effects (Hedges *g =* − 0.35), showing breathwork lowered psychological stress more than control conditions. Moreover, the authors performed an analysis for the effects of breathwork specifically on levels of anxiety (*n* = 20), which, overall, showed efficacy at improving symptoms with medium effect sizes (Hedges *g* = −0.32). Overall, these findings support the utility of deliberate breathing practices in promoting better mental health (Fincham et al. 2023).

However, effect sizes for breathwork interventions vary widely (Fincham et al. 2023), prompting the question: why? One potential key factor is that current methods titrate therapeutic breathing rates between 5 and 6 breaths per minute using heart rate variability, an index of overall vagal tone (Sevoz‐Couche and Laborde 2022). In the titration procedure, participants perform breathing rates between 4 and 7 breaths per minute, and the breathing rate that induces the highest levels of heart rate variability is used for therapy (Fisher and Lehrer 2022; Lehrer et al. 2000). While effective, this approach overlooks well‐documented interindividual differences in spontaneous breathing patterns (Soroka et al. 2025). Our results suggest that a radically individualized strategy, where breathing exercises are tailored to match each person's unique dynamics, could potentially increase the effectiveness of breathing interventions at reducing stress and anxiety.

Future work could record breathing activity, then, using a machine interface, compute breathing dynamics in real time in order to better account for interindividual differences in breathing patterns. Based on our findings, biofeedback technology could then be used to guide participants to breathe slower, less entropic, and with less complexity relative to their baseline, thereby optimizing their breathing intervention to reduce feelings of anxiety. Put differently, the development of a system that continuously analyzes an individual's breathing dynamics in real time and adjusts biofeedback to match their unique physiological signature could enhance therapeutic efficacy.

## 4 Limitations

Certain limitations must be considered when interpreting our results. Most importantly, while this study examines the relationship between breathing dynamics and levels of anxiety and working memory performance, it does not directly assess the underlying neural mechanisms. Additionally, we acknowledge that there has been significant literature on the effect of the breathing phase on affective and cognitive processes. Unfortunately, the length of our recording periods was relatively brief per condition (~5 min), which precluded us from being able to compute our dynamic measures using the breathing‐phase time series. For example, metrics like sample entropy would require on the order of several hundred breathing cycles to yield stable results (Yentes et al. 2013). Finally, because our data was collected from a subclinical sample, it would be important in the future to compare healthy controls with participants meeting diagnostic criteria for anxiety disorders to clarify how breathing dynamics relate to pathological anxiety.

Future studies should compare healthy versus pathological anxiety and utilize neuroimaging (i.e., EEG, MEG, fMRI) to characterize the strength and direction of the relationship between breathing and brain dynamics and determine how their coupling and alignment to the external environment relate to affective and cognitive functioning. Moreover, each condition's recording length should be extended to capture at least 200 breathing cycles per participant in order to be able to properly measure the dynamics of the breathing phase timeseries.

## 5 Conclusion

Our findings demonstrate that the breathing signal displays patterns of activity over time, which can be quantified by using specific metrics indexing dynamic features that carry behavioral relevance. We show the relative contribution of each of these metrics to the overall variance of the breathing signal in the resting state. Then, we show the task‐based modulation of these metrics; this suggests the potential behavioral relevance of the breathing signals' dynamics. Finally, we show that breathing dynamics contribute in different ways to subjects' anxiety levels and working memory performance. Overall, our findings extend the current understanding of how breathing is related to affective and cognitive processes by highlighting the key role of dynamics in that relationship.

## 6 Methods

### 6.1 Participants

The resting state and BAI data were collected from a sample of 210 participants aged 18–71 (*M* = 23.42, SD = 10.39 years, 141 female). A subset of these participants (*N* = 51) aged 18–25 (*M* = 18.57, SD = 1.36, 36 female) completed the resting state and the working memory paradigm.

Participants were recruited from the Ottawa area and the SONA participant pool at Carleton University in Ottawa, Canada. We used flyers and snowball sampling to advertise our study.

This study was approved by the Carleton University Research Ethics Board‐B (Protocol #118891, #11950, and #117723). The studies were conducted following the Tri‐Council Policy Statement: Ethical Conduct for Research Involving Humans guidelines (TCPS‐2), and all ethical regulations relevant to human research participants were followed.

### 6.2 Breathing Data Acquisition and Preprocessing

Breathing data were recorded during a 5‐min resting state and an approximately 30‐min task state with eyes open using a Vernier GoDirect breathing belt (https://www.vernier.com/product/go‐direct‐respiration‐belt/). The breathing vector was sampled at 10 Hz across the continuous costal contractile distance at the level of the diaphragm.

Breathing data preprocessing was performed using a series of Python modules contained in Neurokit2 (Makowski et al. 2021). The data were first band‐passed with cut‐off frequencies of 0.05 and 3 Hz to remove any high and low‐frequency noise. Peaks and troughs in the breathing vector were then extracted using a zero‐crossing algorithm (Khodadad et al. 2018). Using customized Neurokit2 Python modules, we extracted the breath‐breath interval and peak amplitude time series from the raw breathing signal (Makowski et al. 2021).

We then computed the dynamical features described below for the breath‐breath interval and the peak amplitude time series for statistical inferences. All statistical inferences were performed on log‐transformed data to account for non‐normality (West 2022). All data were harmonized to minimize site differences before analysis (Fortin et al. 2017, 2018).

### 6.3 Quantifying Breathing Dynamics

#### 6.3.1 Mean

A time series' mean value represents its distribution's central tendency (Khorana et al. 2023). The mean was used to represent the average breathing amplitude in arbitrary units, and the breathing rate was quantified in breaths per minute. The mean breathing amplitude and rate were computed using the rsp_analyze() function from the Neurokit2 package (Makowski et al. 2021).

#### 6.3.2 Standard Deviation

The standard deviation of a time series represents its overall standardized variance (Khorana et al. 2023). The SD of the breathing waveform was computed using the rsp_analyze() function from the Neurokit2 package (Makowski et al. 2021).

#### 6.3.3 Coefficient of Variation

The coefficient of variation of a time series provides a standardized measure of variability by dividing the standard deviation by the mean of the time series (Ospina and Marmolejo‐Ramos 2019). CV provides a normalized SD in the case where the means of two signals are different, for example, in the case of the breathing rate and amplitude time series. The CV of the breathing waveform was computed using the rsp_analyze() function from the Neurokit2 package (Makowski et al. 2021).

#### 6.3.4 Autocorrelation Window

The autocorrelation function (ACF) quantifies the degree of similarity between a time series and a lagged version of itself over shorter and longer time intervals (Wolman et al. 2023). ACF is calculated by correlating a signal with itself at different lags, indicating the time intervals where the signal stays self‐similar. To parameterize ACF, the lag where ACF drops below 0.5 is extracted (Honey et al. 2012). The aforementioned lag is called the autocorrelation window 50 (ACW). The autocorrelation function was computed using the signal_autocor() function from the Neurokit2 package (Makowski et al. 2021).

#### 6.3.5 Power Spectral Density Slope

The power spectral density (PSD) quantifies how the power of a signal or time series is distributed over its frequency range, which for breathing typically ranges between 0 and 0.5 Hz (Kluger, Gross, and Keitel 2024). The PSD of physiological signals usually has a power‐law relationship between the frequencies and power (Barnsley et al. 1988; Eke et al. 2002; He 2014). This power‐law relationship is mathematically expressed as $\mathrm{P\alpha}\frac{1}{f^{\beta }}$ where *P*, f and β represent power, frequency and power‐law exponent, respectively. Taking the logarithm of both sides makes the relationship linear. Afterwards, one can perform a linear regression to estimate β, which is the slope of the PSD in log‐scale. Decreases in the PSD slope represent a shift in the power distribution towards faster frequencies, while increases in the PSD slope represent shifts towards slower ones. The PSD slope was computed using the fractal_psdslope() from the Neurokit2 package (Makowski et al. 2021).

#### 6.3.6 Lempel‐Ziv Complexity

Lempel‐Ziv complexity (LZC) evaluates the number of unique patterns of consecutive digits embedded within a time series. The algorithm first binarizes the signal based on whether each time point is above (1) or below (0) the mean. The algorithm then reads the string from left to right and adds a new entry to its memory every time it discovers a substring of consecutive digits it has not previously encountered. The length of the compiled list represents the size of the pattern space, quantified as “bits,” that is necessary to explain the signal (Amigó et al. 2004; Lempel and Ziv 1976; Zhang et al. 2009). In signal processing, signals with a lot of regularity yield a smaller pattern space and thus have low LZC. Conversely, time series that are more irregular will have higher Lempel‐Ziv complexity. Lempel‐Ziv complexity was used to quantify the size of the pattern space associated with the rate and amplitude time series. The LZC was computed by using the complexity_lempelziv() function built within the Neurokit2 package (Makowski et al. 2021).

#### 6.3.7 Sample Entropy

Sample entropy (SampEn) quantifies the irregularity of a system. Calculating sample entropy involves four key input parameters that are the embedding delay (), the embedding dimension (*m*), the tolerance (*r*), and the signal (*N*). The embedding delay () corresponds to the delay in samples between the original signal and its delayed versions, which you wish to compare. The embedding dimension (*m*) corresponds to the length of the delayed states. The tolerance (*r*) represents the range of pattern‐matching acceptability of the signal with embedded versions of itself and is operationalized as a percentage of the standard deviation of the time series with length *N*. Overall, sample entropy (, *m, r, N*) measures the probability that a sequence with length *m*, number of time‐points between its elements, and within time series of length *N*, will remain similar to the subsequent *m* + 1 sequence within a threshold of similarity, *r*.

The SampEn was used to quantify the degree of irregularity of the rate and amplitude time series, whereby higher levels of sample entropy represent higher levels of irregularity in the breathing signal (Richman and Moorman 2000). The SampEn was computed using the entropy_sample() function from the Neurokit2 package (Makowski et al. 2021).

#### 6.3.8 Multiscale Entropy

Multiscale entropy (MSE) quantifies the sample entropy of a time series computed across different timescales and, despite its name, is a measure of complexity (Costa et al. 2002, 2005; Hutcheon et al. 2023; Lee and Choi 2018). The researcher defines the timescales analyzed in multiscale entropy by downsampling the original time series at longer time resolutions operationalized as the scaling factor tau (τ). At scale 1, the time series is the raw signal itself. At the value “n” of τ, the length of the coarse‐grained time series is determined by dividing the length of the original time series by τ. In other words, the coarse‐grained signal used for computing MSE is derived from the original signal by averaging non‐overlapping segments of size τ. MSE was computed using the entropy_multiscale() function from the Neurokit2 package (Makowski et al. 2021).

### 6.4 The Beck Anxiety Inventory

Aaron Beck and colleagues developed the Beck Anxiety Inventory to measure the severity of anxiety symptoms (Steer and Beck 1997). The Beck Anxiety Inventory is a self‐report scale that consists of 21 items, each describing a common symptom of anxiety such as nervousness, fear of losing control, or heart pounding. Respondents rate the intensity of each symptom over the past month on a scale from 0 (not at all) to 3 (severely—It bothered me a lot). The Beck Anxiety Inventory has been validated in a wide range of clinical settings, which makes it a suitable tool for quantifying levels of anxiety in this study (Benuto et al. 2020; Chapman et al. 2009; Lee et al. 2018; Schouten et al. 2020). Descriptive statistics for the Beck anxiety scores used in this study can be found in Table S2.

### 6.5 Working Memory Data Acquisition and Preprocessing

**FIGURE 5:**
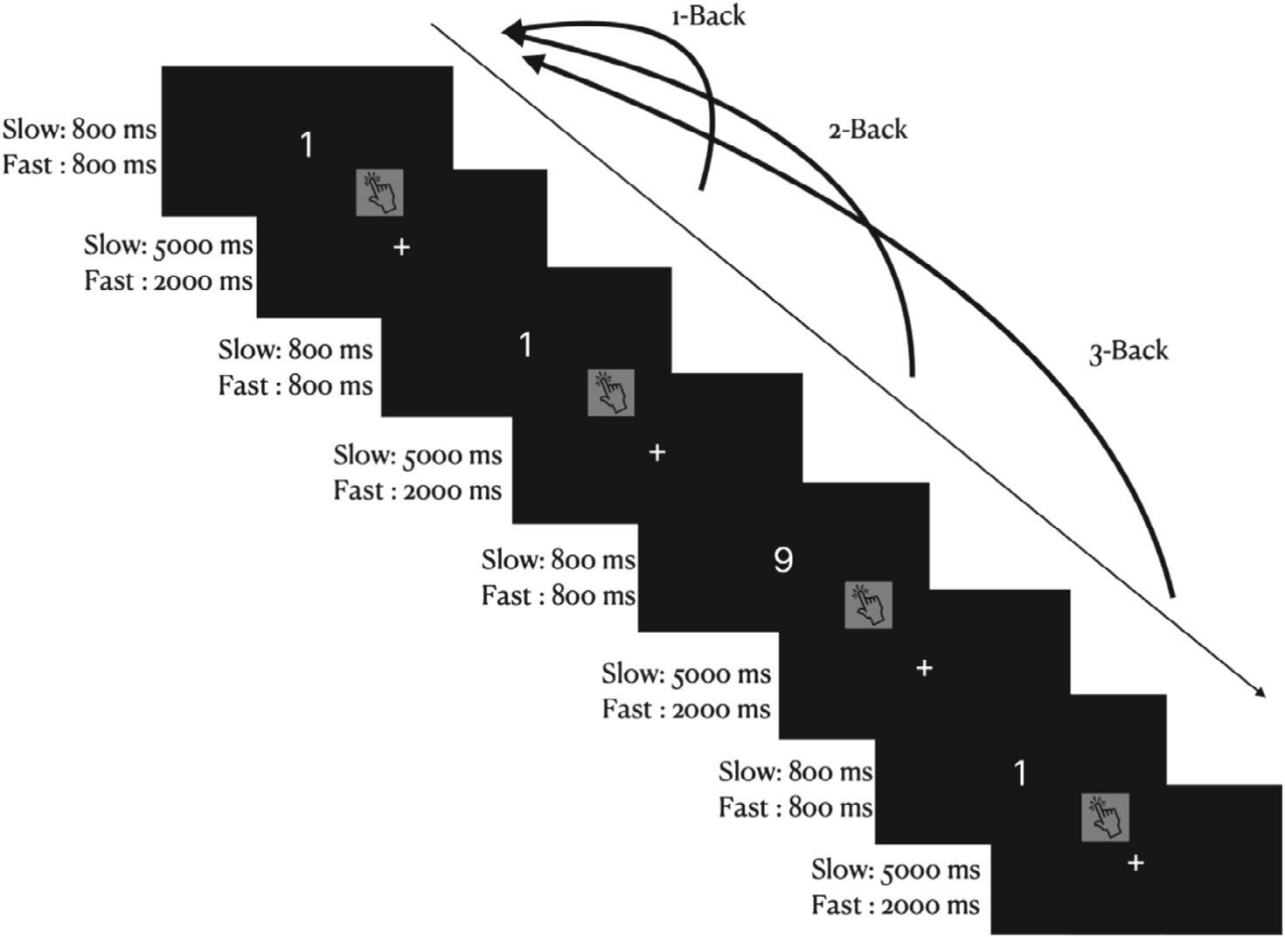
Schematic representation of the N‐back task. The N‐back involves presenting the participant with a sequence of 80 numerical stimuli (0–9). The stimuli were presented separately in the center of a gray screen for 800 ms. Each stimulus was separated by a time lag of either 2000 or 5000 ms, called the intertrial interval (ITI). Using a repeated measures block design, numbers appeared and disappeared on their screen. Participants indicated if the number they currently see on their screen is a recurrence of a number previously seen “N” trials ago. Participants gave responses (yes/no) for each trial using a button click. Which number that the participant is monitoring for the recurrence will depend on the load of the task. This paradigm consisted of three loads. Using a block design, participants will be asked to indicate if the current number they see on their screen is a recurrence of a number presented one, two, or three trials before (1‐, 2‐, 3‐back, respectively). The n‐back lasted approximately 30 min (Cohen et al. 1997).

Participants completed six conditions of a modified N‐back task while their breathing was being recorded. During each condition, participants were presented with visual stimuli (numbers from 0 to 9) in the center of a gray screen for 800 ms. Each stimulus was separated by an intertrial interval of either 2000 ms (fast condition) or 5000 ms (slow condition). As numbers appeared and disappeared on the screen, subjects indicated if the current number they saw on their screen matched the number that they saw “N” trials ago (*N* = 1, 2, or 3). Participants responded (yes match/no match) for each trial using a button click (Figure 5 for a graphical representation of the task design; Cohen et al. 1997). Using a 3 × 2 repeated measures block design, this paradigm consisted of six total conditions defined by three loads (1 back, 2 back, and 3 back) and two speeds (slow and fast). The working memory tasks lasted approximately 30 min.

We acquired the behavioral data using a customized experiment built within Psychopy. We preprocessed behavioral data using a custom Python script and used Pandas data frames for manipulation (McKinney 2010). We extracted key variables such as reaction time (key_resp.rt), accuracy (key_resp.corr), and condition labels (thisCond) for further processing.

As a measure of task performance, we computed an efficiency metric by taking the ratio of accuracy to reaction time (accuracy/reaction time), providing an aggregate measure that captured both response correctness and speed (Liesefeld and Janczyk 2019). The NumPy library (Harris et al. 2020) was used to aggregate the breathing and behavioral data across participants and load conditions (i.e., 1 back, 2 back, and 3 back) before being saved for statistical analysis and modeling. Descriptive statistics for performance on the working memory task can be found in Table S2.

### 6.6 Statistical Reproducibility

#### 6.6.1 Investigating the Latent Structures of Breathing Dynamics: Principal Components Analysis

We performed a principal components analysis (PCA) to identify the underlying structures in the breathing data and to reduce dimensionality. The analysis was conducted using JASP version 0.19.3 (Love et al. 2019).

Four principal components (PCs) were extracted based on eigenvalues greater than 1 (Braeken and van Assen 2017). PCA was conducted using varimax rotation to enhance interpretability by maximizing the variance of squared loadings (Kaiser 1958). The rotated component matrix was examined to determine the primary loadings of each feature on the extracted components.

#### 6.6.2 Comparing Rest and Task Conditions: Paired Samples t‐Tests

We compared breathing measurements across rest and task conditions using paired samples *t*‐tests (Goheen et al. 2024). Repeated measures *t*‐tests are specifically designed to measure change within individuals across two conditions and are thus an appropriate choice for our particular research question (Mishra et al. 2019).

#### 6.6.3 Relating Breathing Dynamics to Emotion and Cognition: Partial Least Squares Correlation

Partial least squares correlation (PLSC) is a multivariate approach that allows for the simultaneous examination of relationships between sets of predictor variables (X) and response variables (Y). Unlike traditional regression methods, which aim to directly estimate the coefficients that best fit the response variable, PLSC focuses on extracting latent variables (LV) that capture the maximum covariance between X and Y. This data‐driven approach is similar to PCA in that it determines orthogonal structures embedded in the data. Unlike PCA, the number of latent structures is constrained by the experimental conditions. Unlike standard univariate analyses that examine any single measure independently, PLS detects sets of variables that covary with the experimental design. Thus, an advantage of PLS is that it identifies patterns in a single analytic step, meaning no correction for multiple comparisons is required (McIntosh et al. 1996; McIntosh and Lobaugh 2004; Tenenhaus 1995).

Each parameter in the X and Y matrices is given a singular value weight determined by singular value decomposition, known as a salience (akin to a component loading in principal components analysis), which is proportional to its covariance on each LV (Spreng et al. 2010).

Next, the bootstrap sampling method is used to calculate standard errors for each metric. The upper and lower bounds of the 95% confidence interval of each variable loading are considered, and values that do not cross 0 are considered reliable (McIntosh et al. 1996; McIntosh and Lobaugh 2004; Tenenhaus 1995).

The overall importance of each latent variable in explaining the data is determined by how much of the cross‐block covariance it explains in the data, which is akin to an effect size. The total cross‐block covariance can be estimated as the ratio of the squared singular values on a given LV to the sum of all the squared singular values on all LVs.

The final step in PLSC involves significance testing of the latent variables. PLSC assesses if the variance explained by a given latent variable is significantly different from what would be expected by a null distribution. We generated a null distribution by re‐computing the latent variables from 1000 random permutations of the original data. The explained variance of the original data is then compared to the null distribution, which indicates the significance of the LV.

When interpreting PLSC results, the effect size of an LV in combination with the results from its permutation test determines the significance and impact of each LV, and each singular value determines the importance of a particular feature on an LV given the data (McIntosh et al. 1996; McIntosh and Lobaugh 2004; Tenenhaus 1995). We performed the PLSC analysis using the behavioural_pls function from the pyls Python package (https://github.com/rmarkello/pyls).

## Author Contributions

**Josh Goheen:** conceptualization, investigation, writing – original draft, methodology, visualization, writing – review and editing, software, formal analysis, project administration, data curation. **Yasir Çatal:** methodology, writing – review and editing. **Imola MacPhee:** writing – review and editing. **Tyler Call:** writing – review and editing. **Cameron Carson:** data curation. **Reem Ali:** data curation. **Rabeaa Khan:** data curation. **Kareen Weche:** data curation. **John A. E. Anderson:** supervision, funding acquisition, writing – original draft, writing – review and editing, conceptualization, resources, methodology, visualization. **Georg Northoff:** conceptualization, writing – original draft, writing – review and editing, supervision, resources, methodology, visualization, funding acquisition.

## Conflicts of Interest

The authors declare no conflicts of interest.

## Supporting information


**Data S1:** psyp70149‐sup‐0001‐Supinfo.docx.

## Data Availability

The data that support the findings of this study are available from the corresponding author upon reasonable request. The source data underlying Figures 2, 3, 4 and Table 1 can be found in the following figshare repository https://doi.org/10.6084/m9.figshare.28447751.v4. *Code Availability*: The code used for preprocessing and analysis for the current study is available at the following GitHub repository: https://github.com/CANALLAB/breathing_wm.git
