# Supplementary material for: It's About Time—Breathing Dynamics Modulate Emotion and Cognition
Source: Psychophysiology. 2025 Sep 11;62(9):e70149. doi: 10.1111/psyp.70149 (PMC12424284; doi:10.1111/psyp.70149)
Supplement: Supplementary file 1 — Data S1: psyp70149‐sup‐0001‐Supinfo.docx. [file PSYP-62-e70149-s001.docx]

| 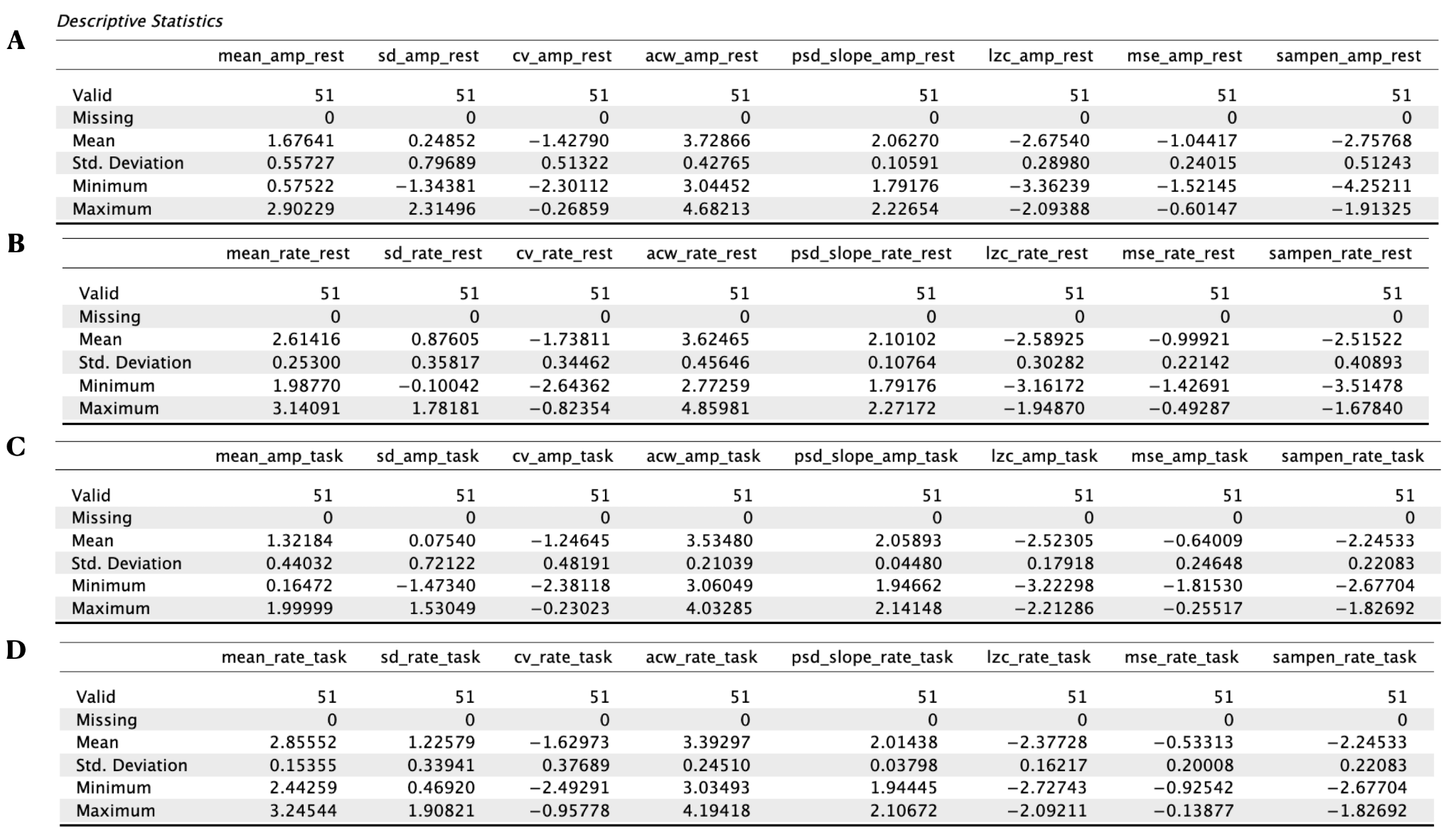 |
| --- |
| **Supplemental Table 1.** Descriptive statistics of breathing dynamics during rest and task conditions. Values represent the log-transformed data. Log transformations were used to account for the non normal normality. LZC = Lempel-Ziv complexity, MSE = multiscale entropy, SampEn = sample entropy, SD = standard deviation, CV = coefficient of variation, ACW = autocorrelation window, PSD slope = power spectral density slope |

| 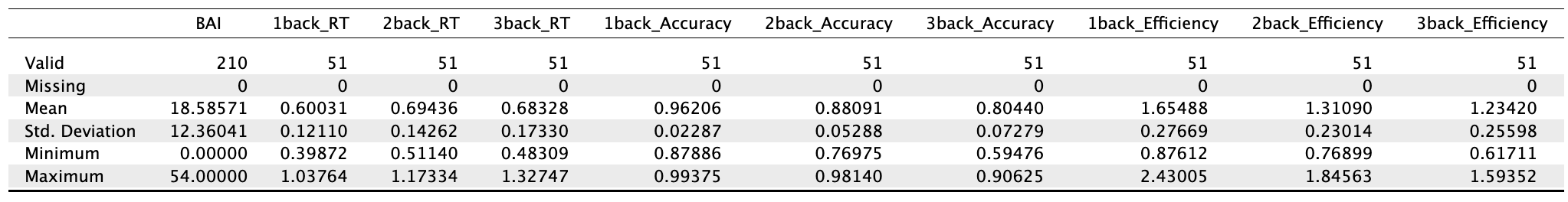 |
| --- |
| **Supplemental Table 2.** Descriptive statistics of Beck anxiety inventory scores across the whole sample and working memory performance for each level of difficulty. |


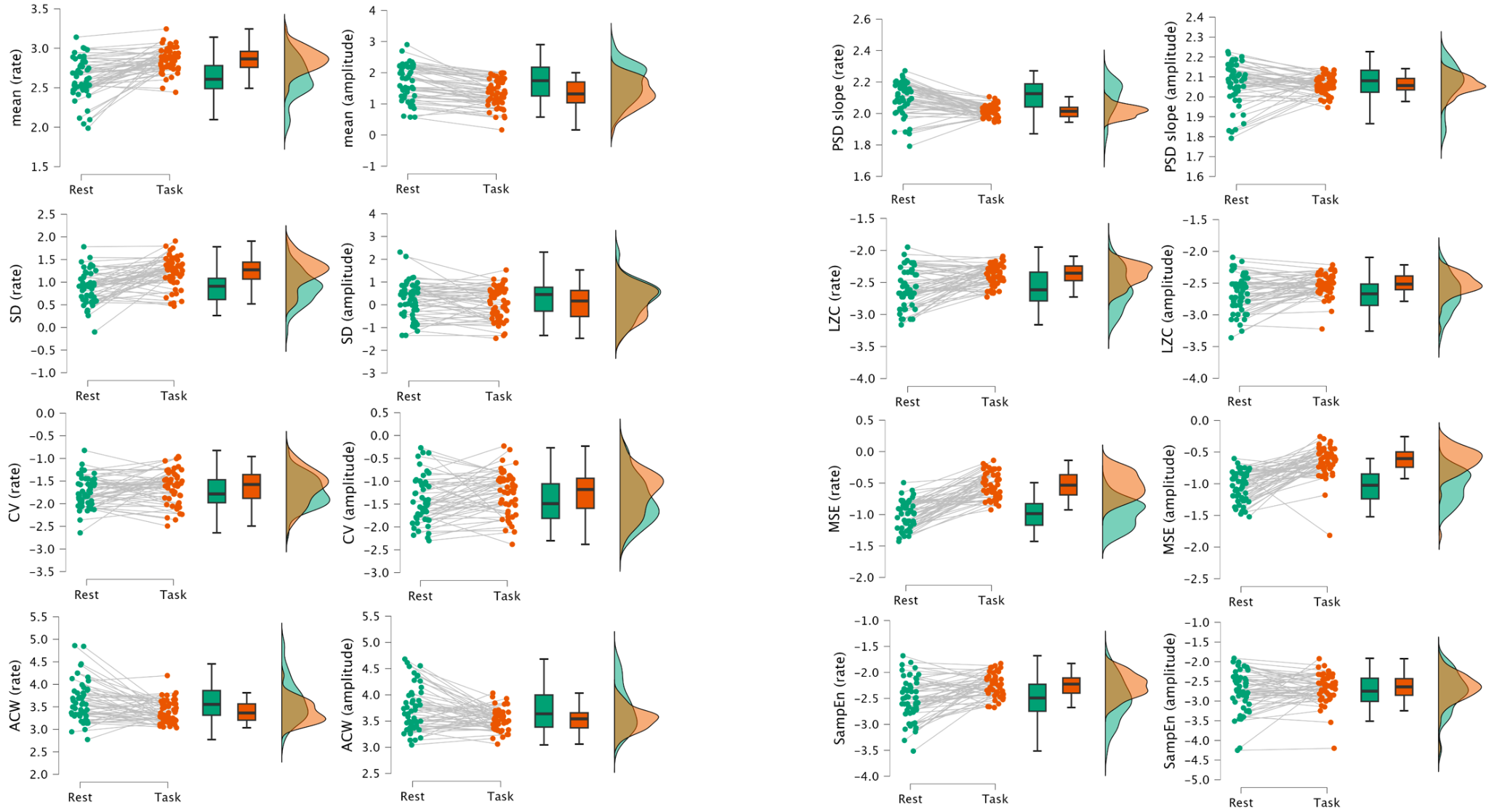


**Supplemental Figure 1.** Raincloud plots representing rest-task changes in breathing dynamics. Values represent the log-transformed data. LZC = Lempel-Ziv complexity, MSE = multiscale entropy, SampEn = sample entropy, SD = standard deviation, CV = coefficient of variation, ACW = autocorrelation window, PSD slope = power spectral density slope


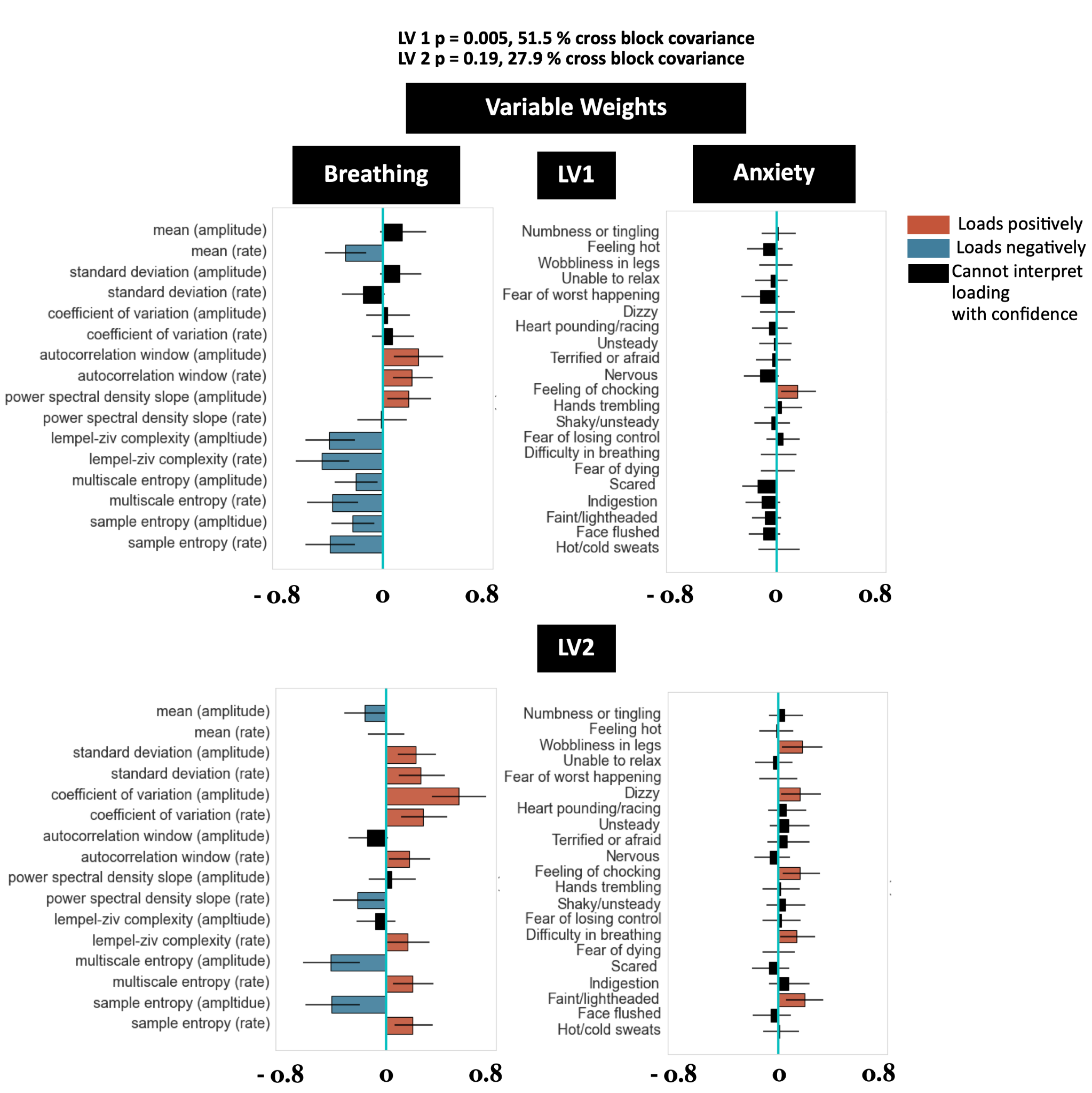


**Supplemental Figure 2.** Unharmonized Partial least squares correlation (PLSC) demonstrating the relationship between breathing dynamics in the resting state and emotion as measured by anxiety levels (i.e., BAI scores) (n=210). Whiskers on the bars represent the 95% confidence intervals of the variable weights assessed from 1000 bootstrap samples.


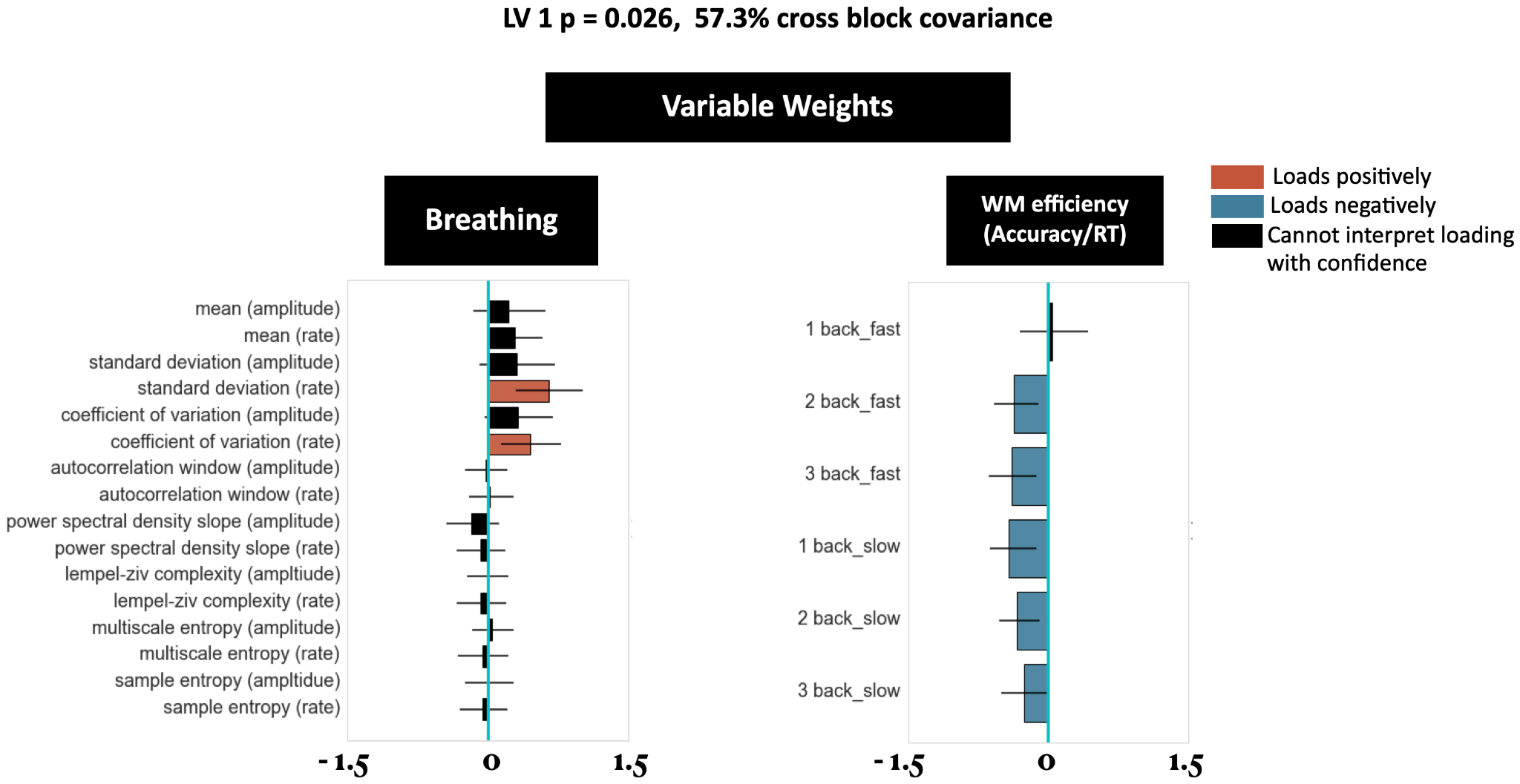


**Supplemental Figure 3.** Partial least squares correlation (PLSC) demonstrating the relationship between breathing dynamics in the task state and cognition as measured by working memory efficiency (accuracy/reaction time) across increasing load conditions (i.e., 1-back fast, 2-back fast, and 3-back fast, 1-back slow, 2-back slow, and 3-back slow; n=51). Whiskers on the bars represent the 95% confidence intervals of the variable weights assessed from 1000 bootstrap samples.


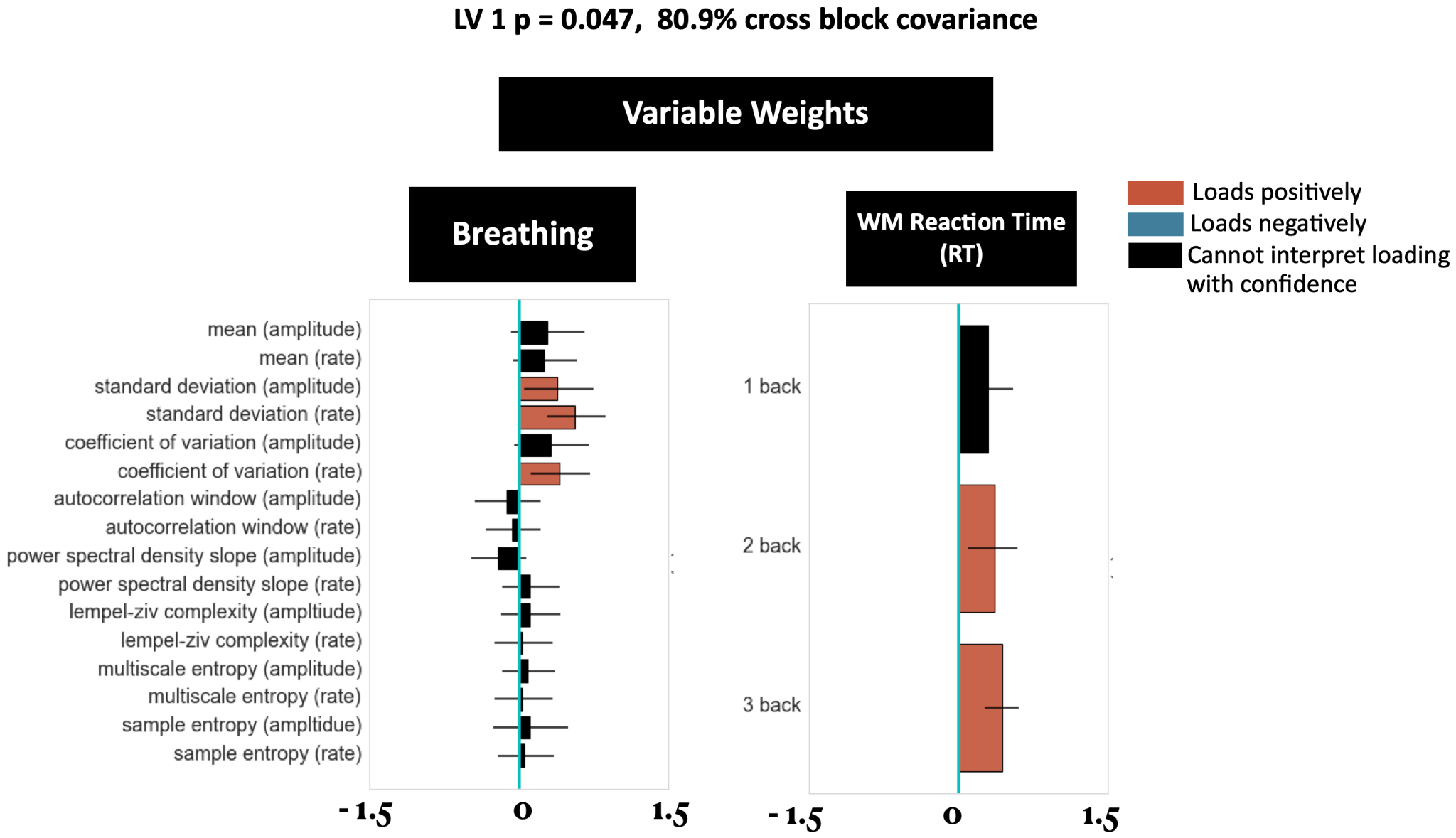


**Supplemental Figure 4.** Partial least squares correlation (PLSC) demonstrating the relationship between breathing dynamics in the task state and cognition as measured by working memory reaction time across increasing load conditions (i.e., 1-back, 2-back, and 3-back; n=51). Whiskers on the bars represent the 95% confidence intervals of the variable weights assessed from 1000 bootstrap samples.


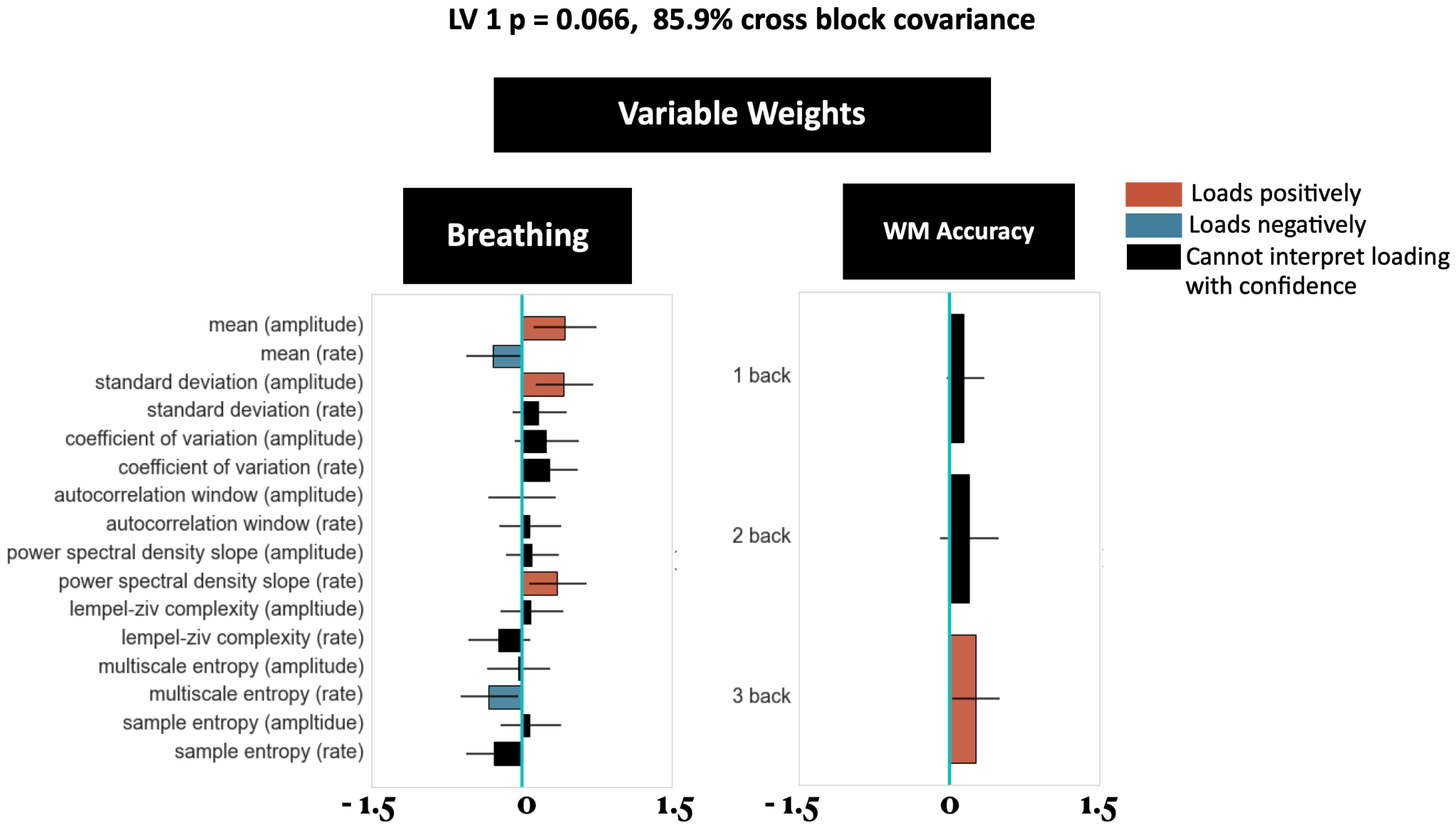


**Supplemental Figure 5.** Partial least squares correlation (PLSC) demonstrating the relationship between breathing dynamics in the task state and cognition as measured by working memory accuracy across increasing load conditions (i.e., 1-back, 2-back, and 3-back; n=51). Whiskers on the bars represent the 95% confidence intervals of the variable weights assessed from 1000 bootstrap samples.

| 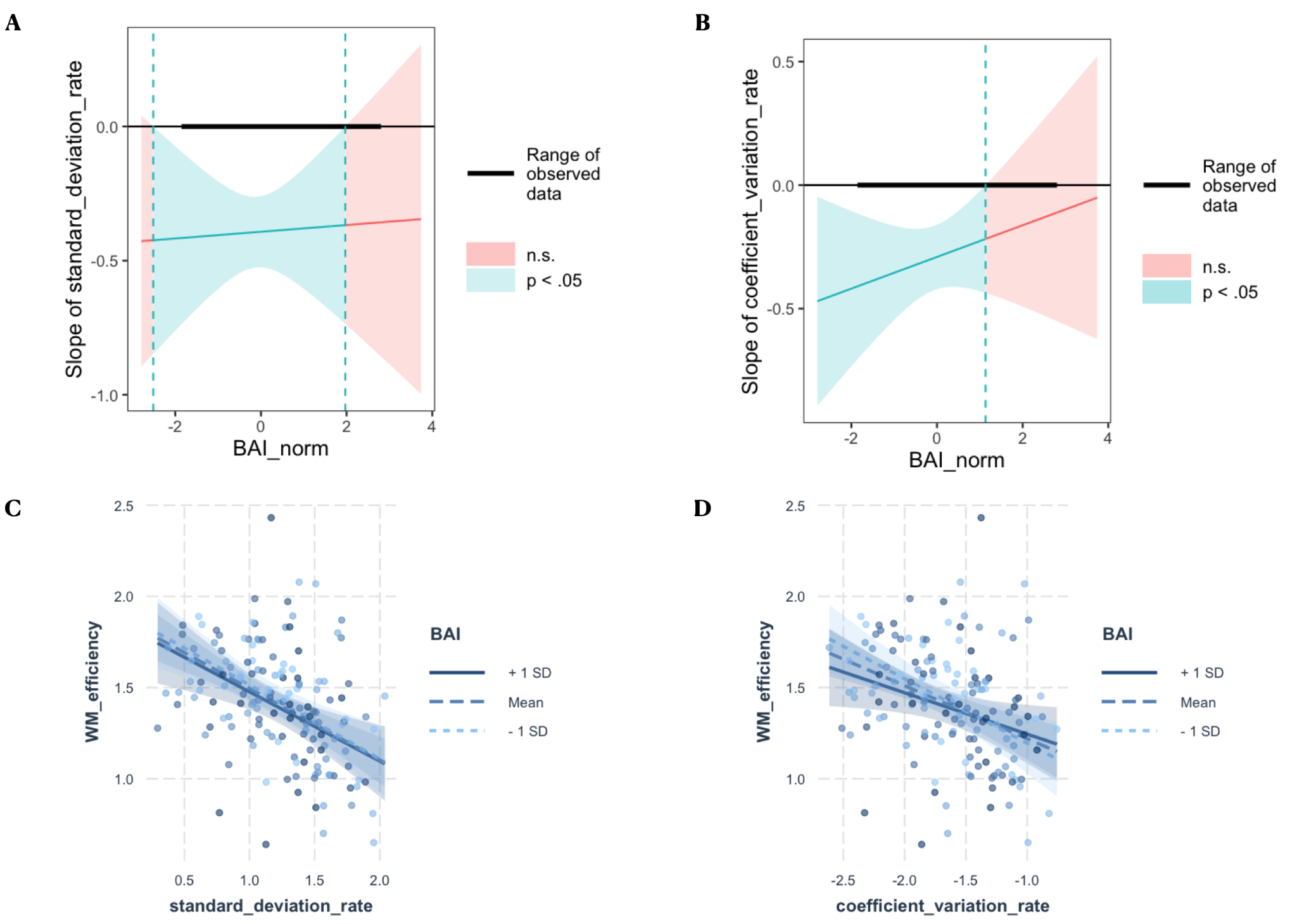 |
| --- |
| **Supplemental Figure 6**. Panels **A** and **B** represent Johnnson-Neyman plots, which identify the specific values of the moderator variable (normalized Beck Anxiety Score; BAI_norm) at which the effect of the predictor on the outcome is statistically significant. Panels **C** and **D** are plots of the interaction between Beck anxiety scores, breathing dynamics, and working memory efficiency (i.e., accuracy/response time). |

| 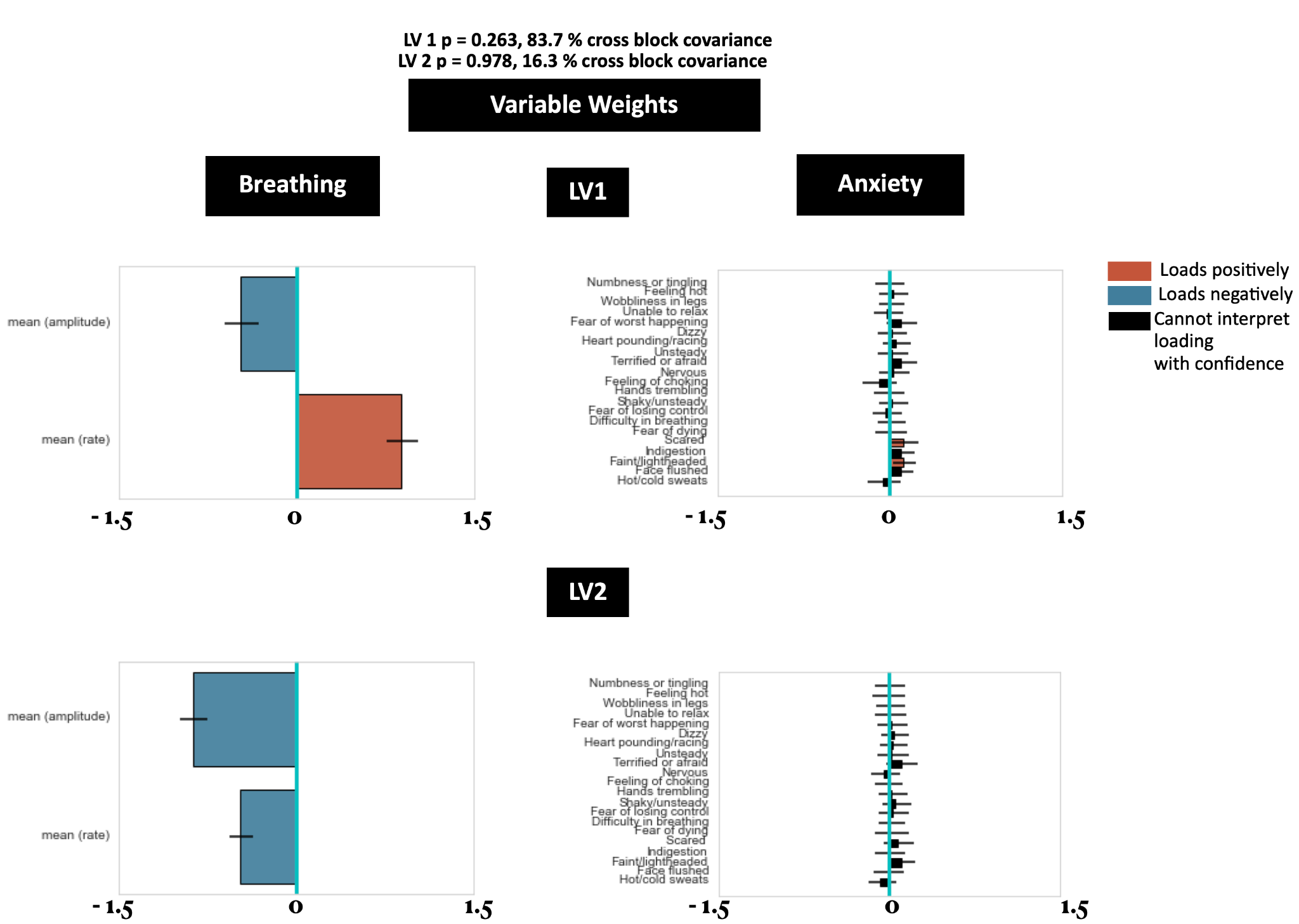 |
| --- |
| **Supplemental Figure 7** Control analysis using only the mean values of the breathing rate and amplitude time series during the resting state to relate to anxiety levels. No reliable LVs were found in this analysis highlighting the importance of dynamics in the relationship between breathing and anxiety levels. |

| 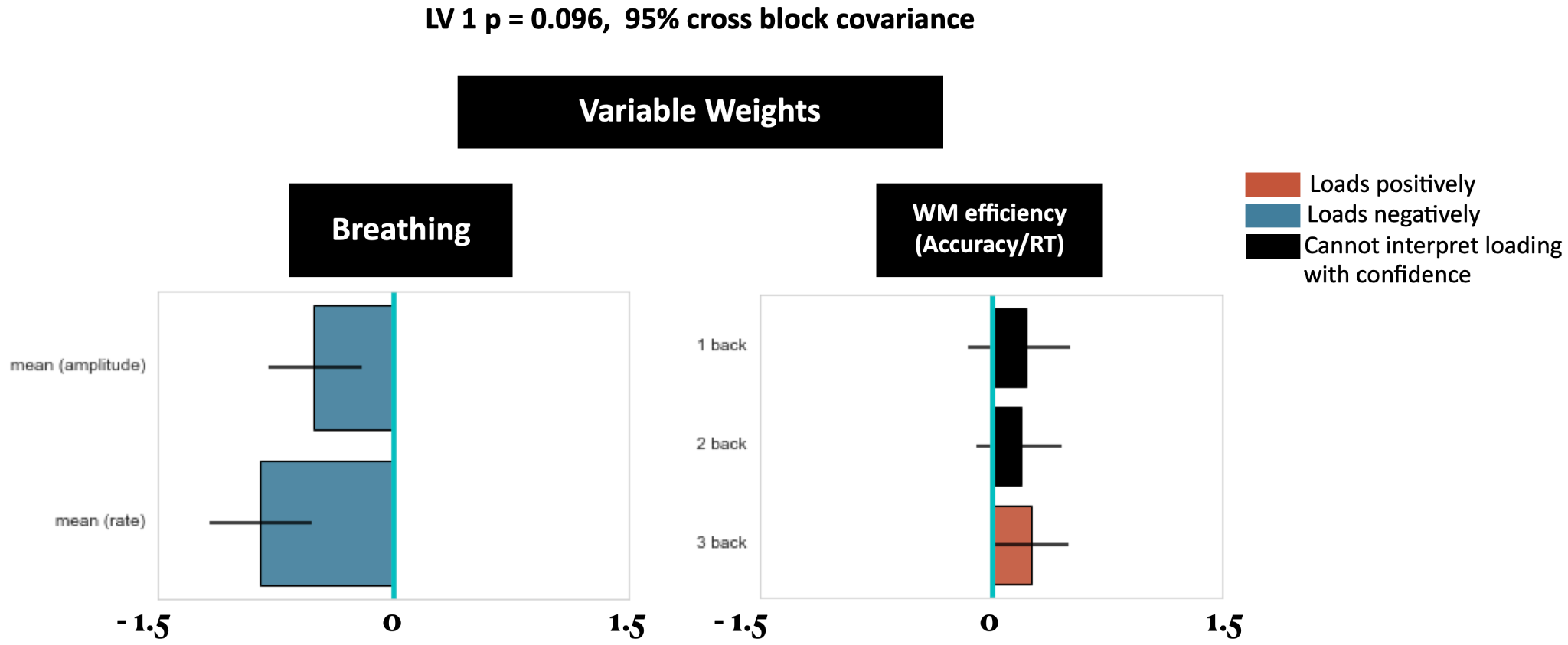 |
| --- |
| **Supplemental Figure 8** Control analysis using only the mean values of the breathing rate and amplitude time series during the *N*-back task to relate to working memory performance. No reliable LVs were found in this analysis, highlighting the importance of dynamics in the relationship between breathing and working memory performance. |

| 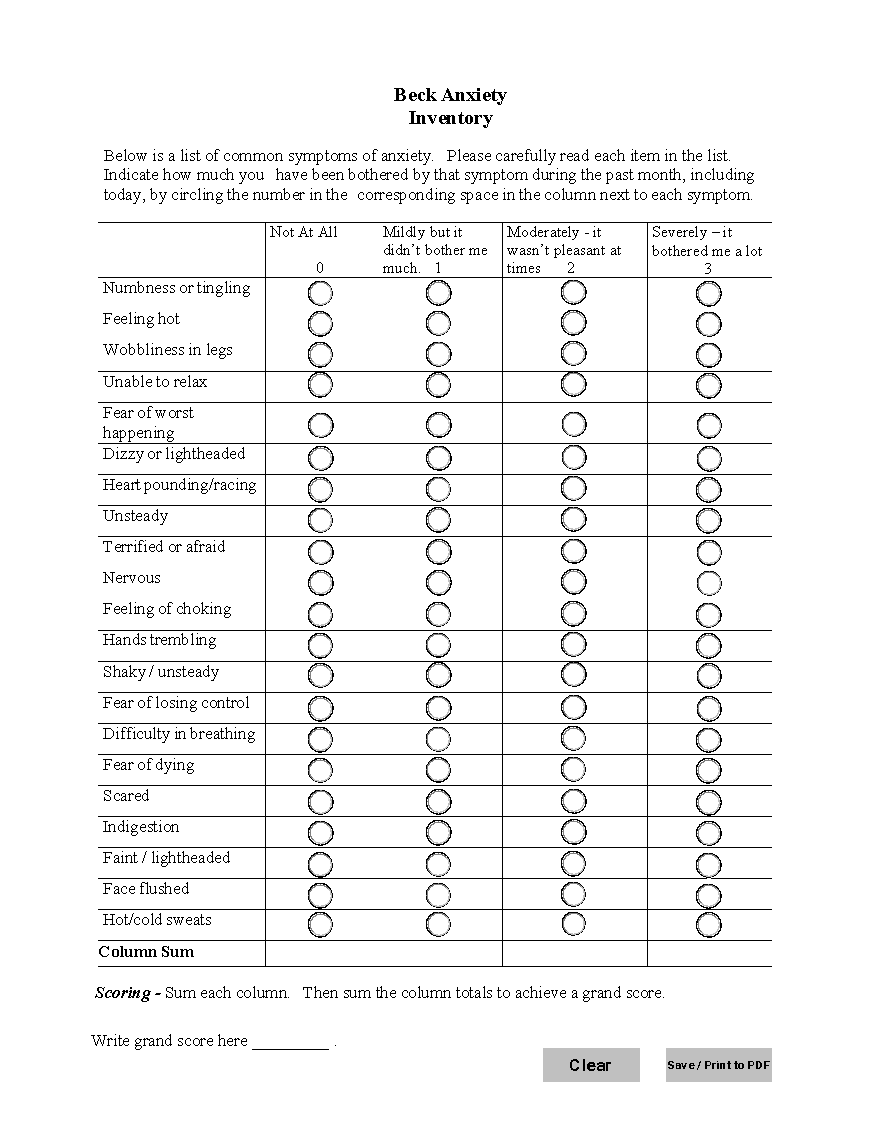 |
| --- |
| **Supplemental Figure 9**. The Beck Anxiety Inventory (Beck et al., 1988) |
